# Characterization of promoters in archaeal genomes based on DNA structural parameters

**DOI:** 10.1002/mbo3.1230

**Published:** 2021-10-28

**Authors:** Gustavo Sganzerla Martinez, Sharmilee Sarkar, Aditya Kumar, Ernesto Pérez‐Rueda, Scheila de Avila e Silva

**Affiliations:** ^1^ Programa de Pós‐Graduação em Biotecnologia Universidade de Caxias do Sul Caxias do Sul‐RS Brasil; ^2^ Department of Molecular Biology and Biotechnology Tezpur University Tezpur Assam India; ^3^ Unidad Académica de Yucatán Instituto de Investigaciones en Matemáticas Aplicadas y en Sistemas Universidad Nacional Autónoma de México Mérida Yucatán México

**Keywords:** archaea, energetic features, structural features, TFBS, transcription

## Abstract

The transcription machinery of archaea can be roughly classified as a simplified version of eukaryotic organisms. The basal transcription factor machinery binds to the TATA box found around 28 nucleotides upstream of the transcription start site; however, some transcription units lack a clear TATA box and still have TBP/TFB binding over them. This apparent absence of conserved sequences could be a consequence of sequence divergence associated with the upstream region, operon, and gene organization. Furthermore, earlier studies have found that a structural analysis gains more information compared with a simple sequence inspection. In this work, we evaluated and coded 3630 archaeal promoter sequences of three organisms, *Haloferax volcanii*, *Thermococcus kodakarensis*, and *Sulfolobus solfataricus* into DNA duplex stability, enthalpy, curvature, and bendability parameters. We also split our dataset into conserved TATA and degenerated TATA promoters to identify differences among these two classes of promoters. The structural analysis reveals variations in archaeal promoter architecture, that is, a distinctive signal is observed in the TFB, TBP, and TFE binding sites independently of these being TATA‐conserved or TATA‐degenerated. In addition, the promoter encountering method was validated with upstream regions of 13 other archaea, suggesting that there might be promoter sequences among them. Therefore, we suggest a novel method for locating promoters within the genome of archaea based on DNA energetic/structural features.

## Graphical abstract

A structural parametrization of genetic information into physical attributes enables the location of transcription factor binding sites in the archaeal genome. This study poses a novel method for promoter sequence location in putative regulators of archaea.

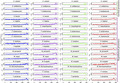

## 1 INTRODUCTION

Archaea represent the third domain of life (Woese, 1987) and include an essential and vast variety of organisms with a large diversity of habitats and lifestyles. This cellular domain has many family divisions belonging to four superphyla: TACK, ASGARD, DPANN, and Euryarchaeota. However, well‐known information is only available for two divisions, Euryarchaeota and Crenarchaeota, the later being a member of the TACK superphylum. In recent years, with the advent of next‐generation sequencing, the availability of archaeal genomes has increased, and more than 300 archaeal genomes have become available to the scientific community, allowing the exploration of diverse functional and evolutionary mechanisms, such as membrane origin, operon organization, and the proteins devoted to regulating gene expression. Nevertheless, there is a lack of well‐annotated archaeal genomic data (Zuo et al., 2015), which enables a lush path toward genomic annotation such as regulatory sequences validation.

The transcription of DNA into RNA and its regulation are central processes in the genetic information flux. Research accumulated in the last few years has evidenced that transcription in archaeal organisms can be roughly described as a simplified version of its eukaryotic relatives (Gehring et al., 2016). The initiation process begins with the binding of a TATA‐binding protein (TBP) and a transcription factor B (TFB) to a specific DNA segment, defined as a promoter, allowing the recruitment of the RNA polymerase (RNAP) enzyme. Additionally, the initiation might be optimized with the presence of a transcription factor E (TFE) protein (Ao et al, 2013). Subsequently, an open complex is assembled, followed by the elongation process whereby the RNAP carries out the synthesis of a messenger RNA molecule (mRNA) (Smollet et al., 2017; Soppa, 1999). In general, three main conserved DNA elements devoted to the transcription process have been identified as common to all archaeal groups: (i) an initiator element (INR) around the transcription start site (TSS); (ii) the TATA box element, centered around −26/27 relative to the TSS; and (iii) an element upstream the TATA box comprising two adenines at −34 and −33, which is designated as “transcription factor B recognition element” (BRE). These elements, INR, TATA box, and BRE, are crucial to initiating transcription in archaeal genes. They also present a close homology to eukaryotic transcriptional machinery (Gehring et al., 2016; Soppa, 1999).

An in‐depth analysis of archaeal promoter elements will provide comprehension of the gene functionality. As an example, there are advances in biotechnology that have employed promoter identification tools to enhance gene regulation and optimize biological processes. The broader comprehension of promoter activity could, in theory, enable full control over the start and halt of the expression of specific genes (Kernan et al., 2017). The production rise in biosynthetic processes is related to the control of regulatory pathways (Ren et al., 2020). For example, clinical biology has benefited from promoter identification due to the increased mutation rate found in regulatory regions that may lead to antibiotic resistance. Evolutionary biology has also applied promoter identification as part of the process to understand better horizontal gene transfer between species of the three domains of life (Khademi et al., 2019).

Bioinformatics tools employ physical assets of the genetic material and relate these with gene expression variance, enabling the distinction of specific regions such as promoters. The study of DNA structural features may give rise to more information about promoter activity than a primary sequence analysis (Bansal et al., 2014; de Avila e Silva et al., 2011; Kanhere & Bansal, 2005; Yella & Bansal, 2017). Indeed, comparative analysis of bacterial and eukaryotic promoters has shown that Pribnow and TATA boxes, respectively, differ at structure and sequence level from other random locations within and around the promoter (de Avila e Silva et al., 2011; Yella et al., 2018).

When converted into numeric attributes, genetic information will promote enough sensibility for capturing even the smallest alterations among the nucleic acids (Benham, 1996). Hence, we consider the nucleotide conservation found in archaeal promoters (Gribaldo & Brochier‐Armanet, 2006; Londei, 2005) will convey a sustained structural parametrization, enabling the characterization of archaeal promoters. In this work, we selected four structural parameters, namely, DNA duplex stability, enthalpy, curvature, and bendability, which are fundamental in understanding the molecular recognition that happens at a structural level (Ryasik et al., 2018).

## 2 DATASETS AND METHODS

### 2.1 Archaea promoter sequences

To determine the nucleotide composition, a total of 3630 promoter sequences of three archaeal organisms were evaluated, which are divided into 1340 sequences of *Haloferax volcanii* (Babski et al., 2016), 1248 of *Thermococcus kodakarensis* (Jäger et al., 2014), and 1042 of *Sulfolobus solfataricus* (Wurtzel et al., 2009). These particular archaea were selected because they are model organisms and well‐studied members of *Halobacteriales*, *Thermococcales*, and *Sulfolobales*, respectively. They also have available transcriptome data (RNAseq), enabling the possibility of retrieving promoter sequences from their published information. Internal and antisense promoters from the transcriptome dataset were not included due to the limitation of data.

The original data covers 1000 nucleotide length sequences, which contains experimentally identified promoters with their transcription start site (TSS), spanning from −500 to +500. Only primary TSS (pTSS) was considered, a category that accounts for abundant transcripts from this original dataset. A shorter sequence was selected, located at 80 nucleotides upstream and 20 nucleotides downstream of the TSS, that is, the core promoter. This briefer region was chosen because it contains the core promoter element (Aptekman & Nadra, 2018; Haberle & Stark, 2018; Kadonaga, 2012). Accordingly, the core promoter has been detailed as sufficient to convey archaeal and eukaryotic transcription (Bartlett et al., 2000; Haberle & Stark, 2018; Zuo et al., 2015). Indeed, promoters from halophilic archaea were reported to be located in the range proposed here; their TATA boxes were found in a median distance of 31 base pairs (bps) upstream the TSS (Babski et al., 2016). Additionally, 96% of the pTSS TATA boxes from *T*. *kodakarensis* are located in a median distance of 30 base pairs upstream of the TSS (Jäger et al., 2014). The TATA boxes identified in *S*. *solfataricus* were found in a median length of 35 base pairs upstream of the TSS (Le et al., 2017). Each archaeal promoter sequence had a shuffled version assigned to have a control sequence. The shuffling process was performed by the Supplementary Script S4 (https://doi.org/10.5281/zenodo.5137597).

Moreover, upstream regions from 13 other archaea found in the RSAT Prokaryote Database (Nguyen et al., 2018) were selected to validate the method formulated upon the experimentally verified promoters. *Aciduliprofundum boonei* (741 sequences), *Archaeoglobus fulgidus* (866 sequences), *Ferroplasma acidarmanus* (430 sequences), *Haloarcula marismortui* (1998 sequences), *Methanocaldococcus jannaschii* (1866 sequences), *Methanosarcina mazei* (822 sequences), *Methanospirillum hungatei* (1467 sequences), *Methanothermobacter thermautotrophicus* (1870 sequences), and *Pyrococcus furiosus* (1286 sequences) were selected as members of Euryarchaea. The following members of TACK archaea were selected: *Caldivirga maquilingensis* (1669 sequences), *Hyphertermus butylicus* (764 sequences), *Ignicoccus*. *hospitalis* (1005 sequences), and *Thermofilum pendens* (1926 sequences). DPANN and ASGARD archaea were not included due to their data unavailability. These particular organisms were selected because of their key role in the evolution of archaea, posing as unique organisms in the archaeal tree of life (Williams et al., 2017).

### 2.2 Conversion in structural parameters

To convert the DNA sequences into structural parameters, four DNA structural features were selected, namely DNA duplex stability (DDS), enthalpy contribution, bendability, and intrinsic curvature. These features are biologically relevant to characterize promoter regions since they convert DNA information into numeric attributes (Benham, 1996). These four parameters have previously been used and reflect in capturing specific signals that are not evident at the sequence level (Bansal et al., 2014; de Avila e Silva et al., 2011; Kanhere & Bansal, 2005; SantaLucia & Hicks, 2004; Yella & Bansal, 2017; Yella et al., 2018). Moreover, the appointed features can be described as:

- The DDS of double‐stranded DNA is calculated as the sum of its base‐pair free energy. It considers the free‐energy values associated with the 16 possible combinations of dinucleotides (Kanhere & Bansal, 2005).
- Enthalpy parameters refer to thermodynamic processes that occur at a cellular level (e.g., chemical bonds, mass transport inside and outside the cell, and heat spawning) that affect the thermostability of the cell (Privalov & Crane‐Robinson, 2018). These numeric parameters have been taken from DNA melting studies (SantaLucia & Hicks, 2004).
- DNA bendability is a sequence‐dependent measurement, reflecting in the DNA bending itself because of the effect specific proteins have in the molecule's helical structure. By this means, DNA bending facilitates the assembly of transcription complexes (Leonard et al., 1997). TATA's bend angle is wider than GC‐rich sequences; for instance, TA dinucleotides angle the DNA at 6.74°, the most impactful of the 16 dinucleotide combinations (Karas et al., 1996).
- Finally, intrinsic curvature reflects the capacity of DNA to form small circles around its helical axis (Bolshoy et al., 1991). To this end, we used a model based on DNA gel retardation values (BMHT) for its sensibility toward AT‐rich sequences (Bolshoy et al., 1991; Kanhere & Bansal, 2003). BMHT calculation estimated 16 roll and tilt wedge angles based on independent gel mobility experiments performed on a training set of 54 different sequences (Bolshoy et al., 1991).

All the four features selected are sequence‐dependent and their combination yields more information gathered on a sequence (Ryasik et al., 2018). The complete set of promoter sequences was converted into structural parameters through a self‐developed Python script (Supplementary Script S1: https://doi.org/10.5281/zenodo.5137597) that adopts the numeric parameters available in Table 1, except for intrinsic curvature. The curvature calculation hinges on five nucleotides (instead of di and resulted in 4^5^ (Smollet et al., 2017) possible combinations). The 1024 numeric parameters are the result of BMHT calculations (Bolshoy et al., 1991), and they are available in Supplementary Script S2 (https://doi.org/10.5281/zenodo.5137597).

**TABLE 1:** Enthalpy, stability, and bendability parameters for every possible dinucleotide combination

Dinucleotide	Enthalpy (kcal/mol‐bp−1)	Stability (kcal/mol‐bp−1)	DNA bendability (degrees)
AA	−7.6	−1.00	3.07
AT	−7.2	−0.88	2.6
AC	−8,5	−1.45	2.97
AG	−8.2	−1.3	2.31
TT	−7.6	−1	3.07
TA	−7.2	−0.58	6.74
TC	−7.8	−1.28	2.51
TG	−8.4	−1.44	3.58
CC	−8	−1.28	2.16
CA	−8.5	−1.45	3.58
CT	−7.8	−1.28	2.31
CG	−10.6	−2.24	2.81
GG	−8	−1.84	2.16
GA	−8.2	−1.3	2.51
GT	−8.4	−1.44	2.97
GC	−10.6	−2.24	3.06

The structural properties were computed in a one‐nucleotide sliding window. All promoters were aligned relative to their TSS, and numerical values were averaged to get information in each position.

### 2.3 Classification of conserved TATA and degenerated TATA sequences

To classify the core promoters in conserved and degenerated TATA, the MEME Suite—a motif‐based sequence analysis tool (Bailey et al., 2009) was employed. All the sequences were scanned with MEME, and the motifs identified by it were extracted. A key motif for this research would be located in −27/‐28, so the search was directed to this specific region to capture the TATAs. The following parameters on MEME were used in the organisms *H*. *volcanii* and *T*. *kodakarensis*: *i*) 100 nucleotides sequence length, considering the −80 to +20 region, where the core promoter is located (Haberle & Stark, 2018; Kadonaga, 2012); *ii*) a 0‐order background model generated from the supplied sequences; *iii*) zero or one occurrence (of a contributing motif site) per sequence; *iv*) 8 motifs were located; *v*) the width of the motifs varied between six and eight nucleotides (Hausner et al., 1991). The motif discovery had to follow different parameters in *S*. *solfataricus*, in which the width of the motifs was increased from six to fifteen nucleotides to capture the TATA boxes adequately. Hence, TATA boxes and BRE elements were considered. The combination of these two consensuses was described as a critical feature in *Sulfolobaceae* family transcription (Le et al., 2017).

Afterward, the dataset was classified through a self‐developed Python script (Supplementary Script S3: https://doi.org/10.5281/zenodo.5137597), dividing it into two groups: conserved TATA, those motifs identified by MEME, and degenerated TATA, containing sequences which the previously identified motif was not present.

### 2.4 Statistical tests

Statistical tests were conducted to differentiate the two groups this study hinged on. First, the dataset was found not to be normally distributed through the rejection of the null hypothesis achieved by the Shapiro–Wilk test. Then, to determine if the difference between the groups is significant, the Wilcoxon test was applied. Additionally, the nonparametric Kruskal–Wallis test was conducted to determine the difference between variances in specific organisms. These tests were done in the R programming language in the *stats* package.

## 3 RESULTS

### 3.1 Sequence composition

The nucleotide composition of the three archaeal organisms was evaluated to denote the genome configuration particular to each archaeon. Firstly, the 1000 nucleotide sequences are composed of 33.8% of AT in *H*. *volcanii* DS2, 49.3% in *T*. *kodakarensis* KOD1, and 65.5% in *S*. *solfataricus* P2. Second, the core promoter elements (−80 to +20) in these organisms presented an AT value of 40% in *H*. *volcanii*, 56.8% in *T*. *kodakarensis*, and 71.1% in *S*. *solfataricus*.

### 3.2 Conserved TATA and Degenerated TATA boxes.

**FIGURE A1:**
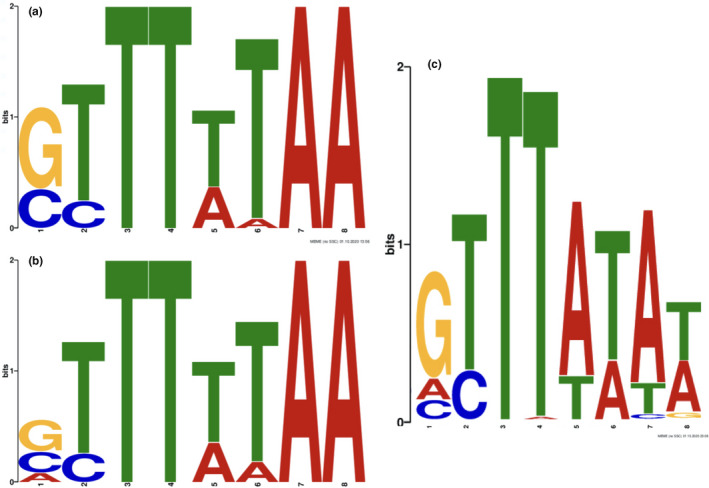
*H*. *volcanii*, *T*. *kodakarensis*, and *S*. *solfataricus* TATA motifs identified by the MEME suite. S1 (a) indicates *H*. *volcanii*, from which the resulting motif was found in a median position downstream of the TSS of 31 bps. The e‐value of this motif is 1.8 e‐092; it has been found in 382 sites; its relative entropy is 12.1 and; information content =13.2. S1 (b) indicates *T*. *kodakarensis*, motifs located in a median distance of 30 bps downstream of the TSS. Its e‐value is 1.3 e‐017; this motif has been located in 257 sites; relative entropy and information content 12.3 and 12.4, respectively. S1 (c) represents the TATA motif found in *S*. *solfataricus* found in a median distance of 30 bps downstream the TSS. The e‐value =6.1e‐022, site count 192, relative entropy =12.5 and, information content =16.2

The datasets were split into two groups to capture particularities and verify the hypothesis of the archaeal transcription being beyond TATA box conservation. The two groups are Conserved TATA and Degenerated TATA. Motifs of eight nucleotides were found in *H*. *volcanii* and *T*. *kodakarensis*. Simultaneously, the outcome of *S*. *solfataricus* encompassed 14 nucleotides. In an attempt to preserve the particularities each archaeon has, the analysis was individually done. The TATA box motif of each organism is found in Figure A1, from where *H*. *volcanii* presented SYTTWWAA, *T*. *kodakarensis* TATA was identified as VYTTWWAA, and *S*. *solfataricus* accounted for KVRWAAA VYTTWWWW motifs.

**FIGURE 1:**
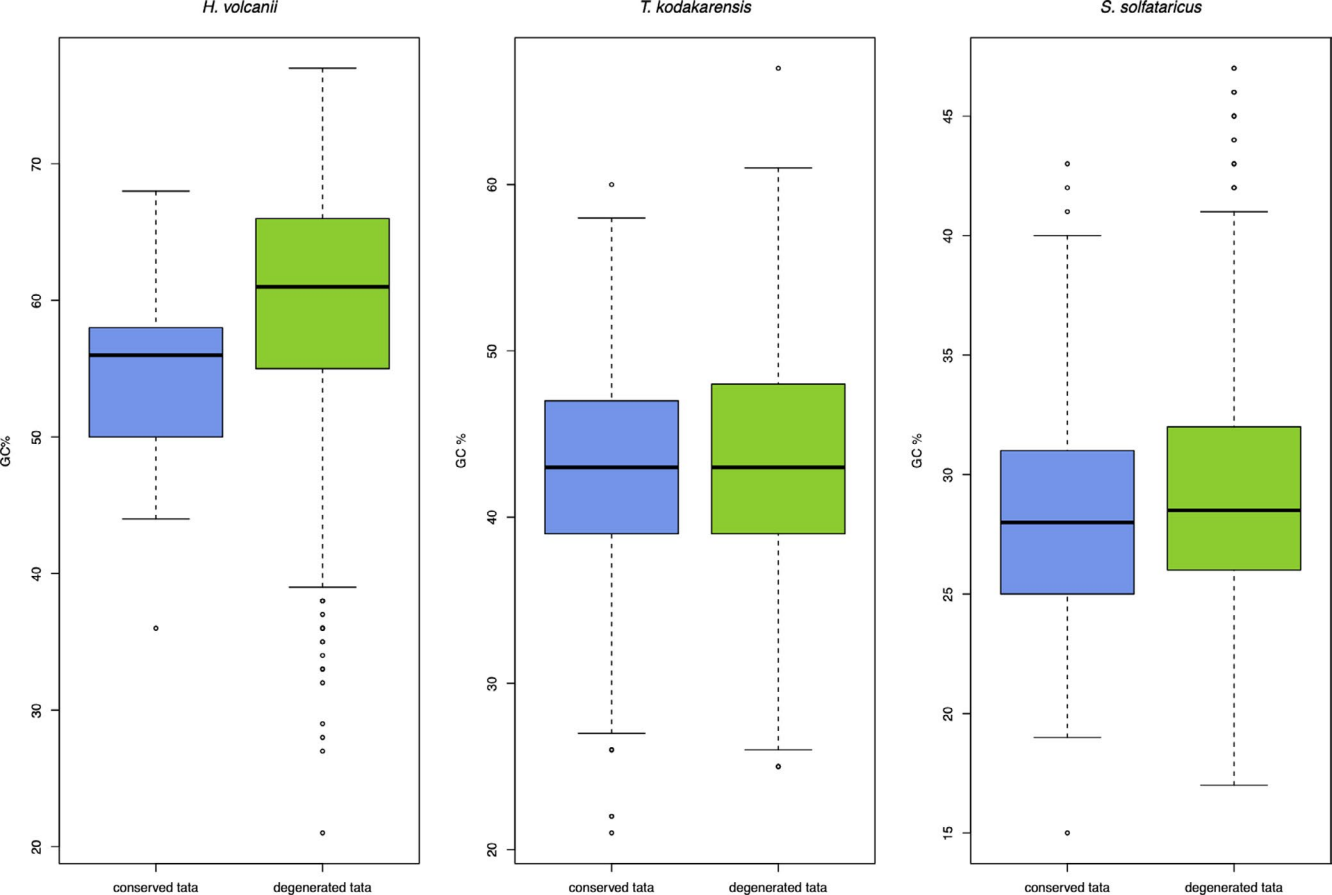
Boxplots of TATA‐containing and TATA‐less promoter sequences in three archaea. We divided 1340 *H*. *volcanii*, 1248 *T*. *kodakarensis*, and 1042 *S*. *solfataricus* sequences into two groups: TATA‐containing and TATA‐less by following Materials and Methods 2.3. Then, we calculated the GC% of each sequence in the groups and created boxplots alongside U tests to discover significance between the groups. The p values in the nonparametric U tests were as follows: 0.0006556, 0.131, and 3.241e‐09 in *H*. *volcanii*, *T*. *kodakarensis*, and *S*. *solfataricus*, respectively

When each one of the motifs was employed to split the dataset, the results of Table 2 were produced. To begin with, 1.56% of 1340 *H*. *volcanii* sequences presented the TATA motif previously identified. The number of sequences containing motifs in *T*. *kodakarensis* was 42.72% and 80.6% in *S*. *solfataricus*. Then, the GC% of each group was evaluated to verify if they yield statistical significance. U tests were performed due to the data not following a normal distribution. Figure 1 shows boxplots from which the means of the conserved and degenerated TATA in *H*. *volcanii*, *T*. *kodakarensis*, and *S*. *solfataricus* are *p* = 0.0006556, *p* = 0.131, and *p* = 0.005365, respectively.

**TABLE 2:** Conserved TATA and degenerated TATA upon core promoter sequences in three archaeal organisms

Organism	Genome GC%	Conserved TATA		Degenerated TATA	
		Number of promoters (%)	GC%	Number of promoters (%)	GC%
*H. volcanii*	66.13	21 (1.56%)	54.09	1319 (98.44%)	60.03
*T. kodakarensis*	50.67	506 (42.72%)	42.55	742 (57.28%)	43.39
*S. solfataricus*	34.48	840 (80.6%)	28.42	202 (19.4%)	30.68

### 3.3 Structural profiles of archaeal promoter sequences vary when transcription factors binding sites

**FIGURE 2:**
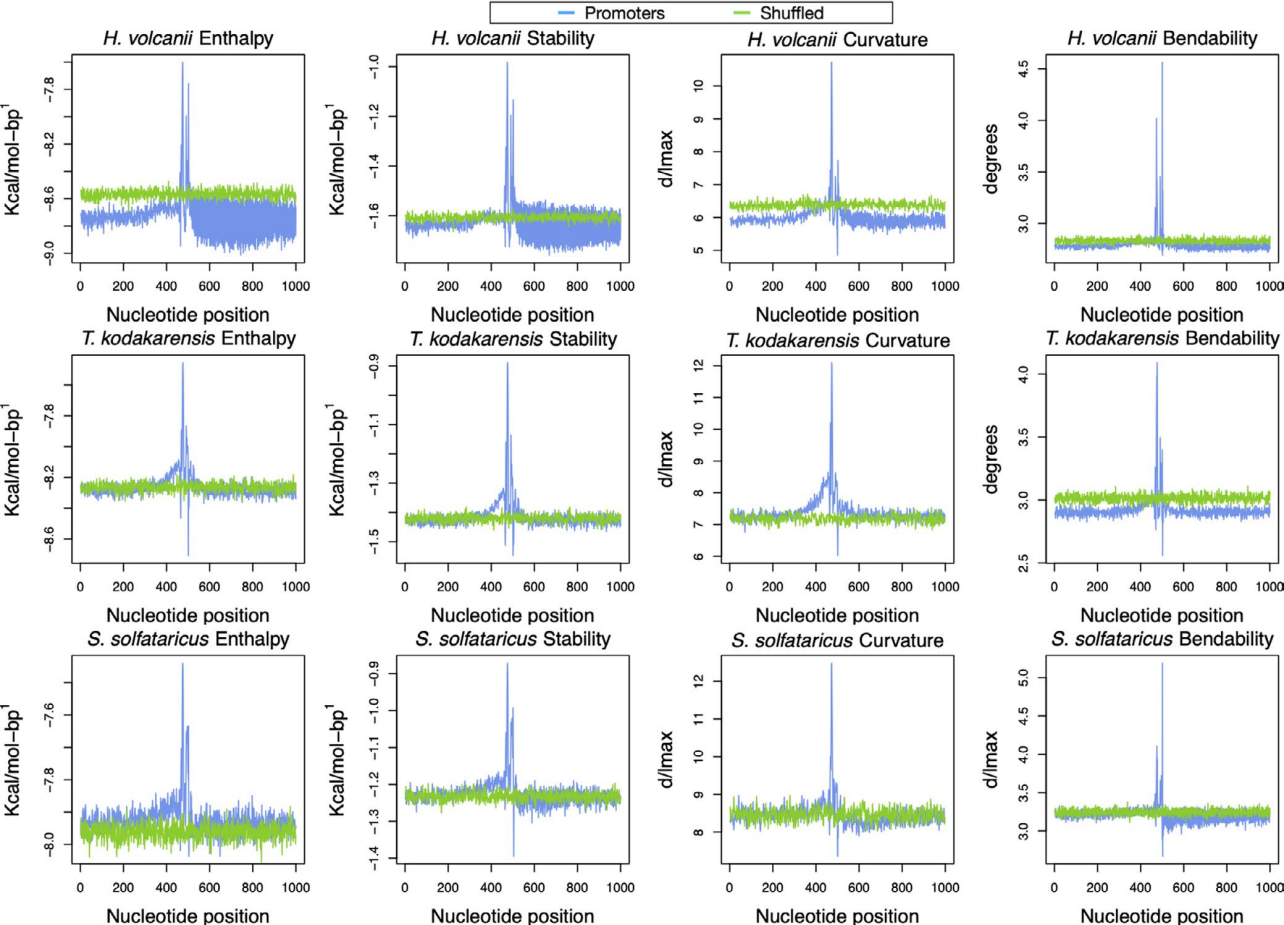
Structural/energetic profiles of 1000 nucleotides found in promoter and shuffled sequences. Energetic/structural features of three archaea. We plotted the average value in each one of the 1000 positions. The highest peak is seen at position −28 in three archaea, four measurements. The blue line represents the promoter sequences and the green line indicates a shuffled version of the promoters. The shuffling process was carried out by a Python script (Supplementary Script S4: https://doi.org/10.5281/zenodo.5137597)

**FIGURE 3:**
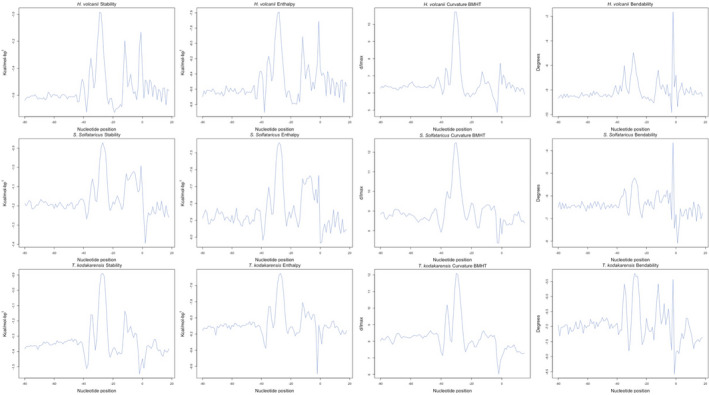
Structural/energetic core promoter profiles. Energetic and sequence‐dependent features of three archaea. We plotted the average of the core promoter positions reported by Kadonaga, 2012; Haberle and Stark, 2018. Our plots indicated a strong signal in i) the TATA box and BRE positions; ii) the PPE area

The entire promoter dataset was converted into enthalpy, DNA Duplex Stability (DDS), bendability, and intrinsic curvature to capture specific signals in wider genome analysis, ranging from −500 to +500. In addition, control sequences were added to elicit the strong signals promoter sequences have (Figure 2). A zoomed version, encompassing the promoter region only, was included in Figure 3, where there is a conserved region around the binding site of the transcription factor proteins: TBP (TATA box, around −28), TFB (BRE, around 2 nucleotides upstream TBP), TFE, whose binding site is located in position −10 (PPE – proximal promoter element) and +1, matching the INR (initiator element).

### 3.4 Definition of a promoter‐like profile

**FIGURE 4:**
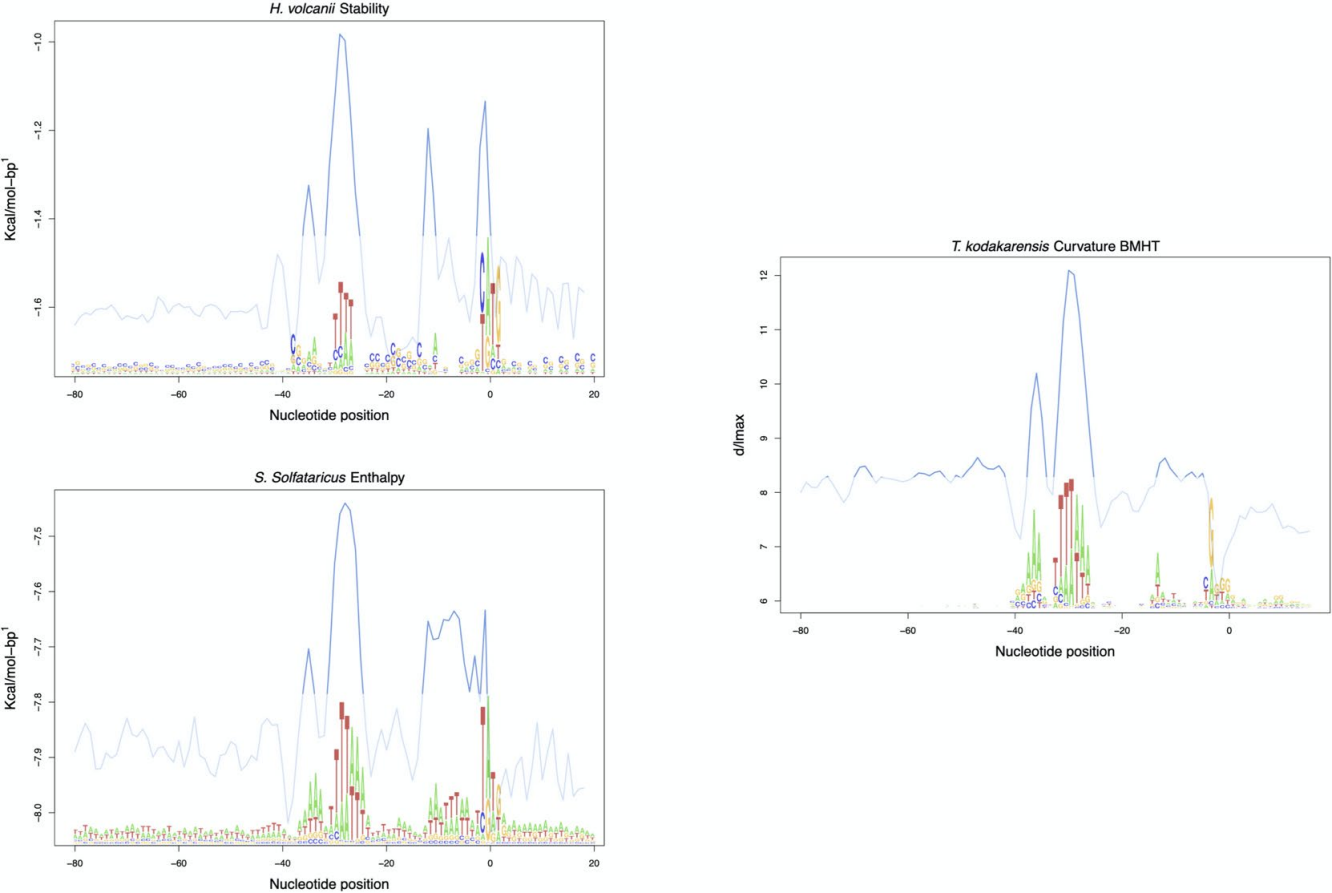
Transcription factor binding sites represented by signals regarding structural/energetic profiles of the core promoter. Nucleotide information (sequence logo profiles) is overlaid with signals that represent the core promoter content

By following the profiles brought by Figure 3, a promoter‐like profile was formed upon the average per position (100 nucleotides) of each feature in the validated promoter dataset. By combining nucleotide information (sequence logo profiles) with the structural parametrization brought by this work, Figure 4 was created. In this, the strong DDS, enthalpy, bendability, and BMHT curvature signals are overlaid with transcription factor binding sites.

### 3.5 Validation of the results with 13 other archaea

**FIGURE 5:**
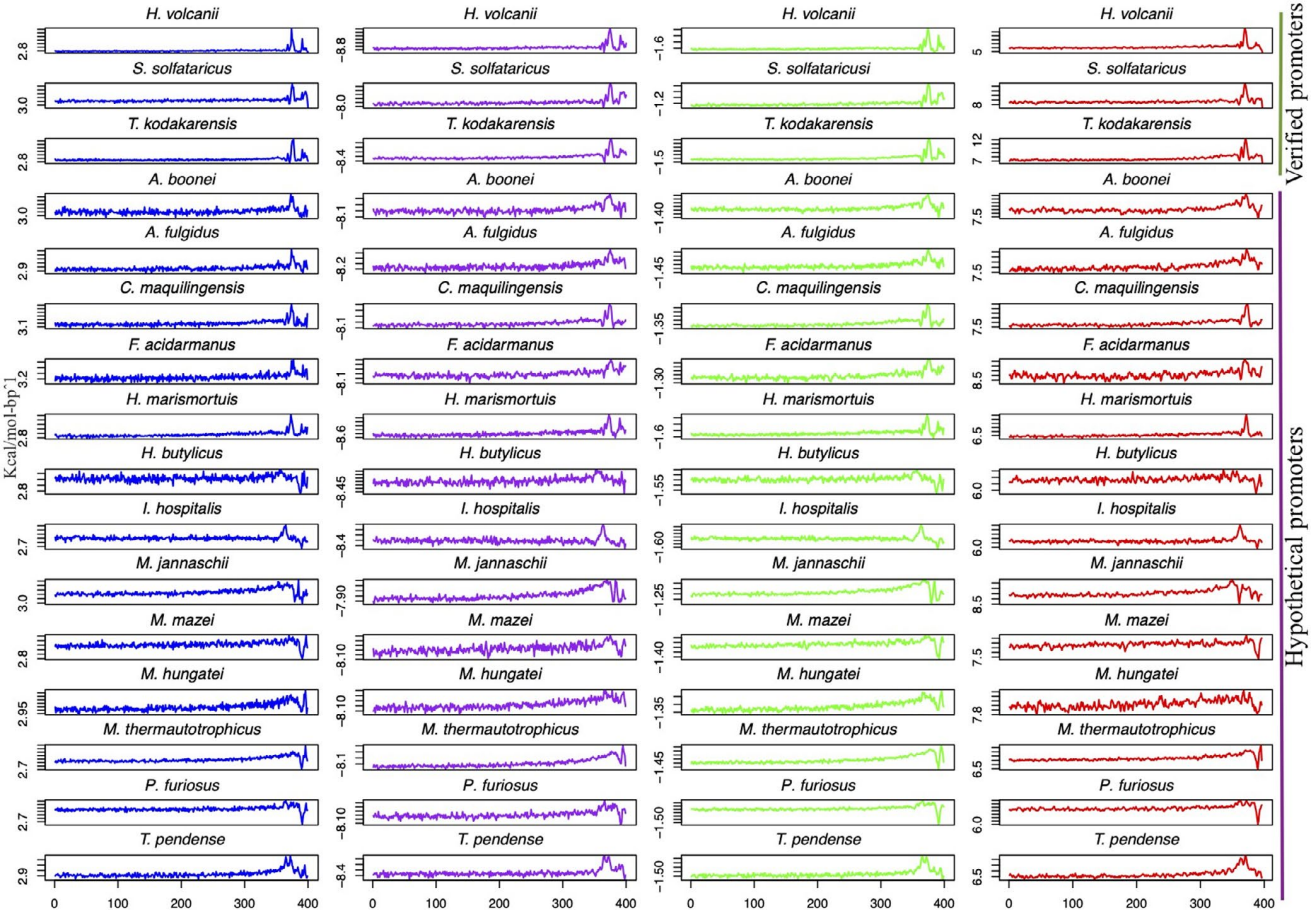
Structural/energetic upstream profiles in thirteen archaea. Thirteen other archaea were selected from 42 to validate the promoter‐like behavior observed. These organisms have 400 nucleotide‐long sequences corresponding to upstream sequences where no annotation toward promoter finding was done. The blue lines represent bendability profiles, the purple enthalpy, the green refers to DDS, and the red is BMHT curvature

Upstream regions of thirteen other archaea divided into four TACKs and nine *Euryarchaea* were included to test the validity of the findings. Figure 5 holds the genomic information of each archaeon plotted against DNA bendability, BMHT curvature, enthalpy, and DDS. In all cases, a strong signal around the ending of the upstream regions was located.

**FIGURE 6:**
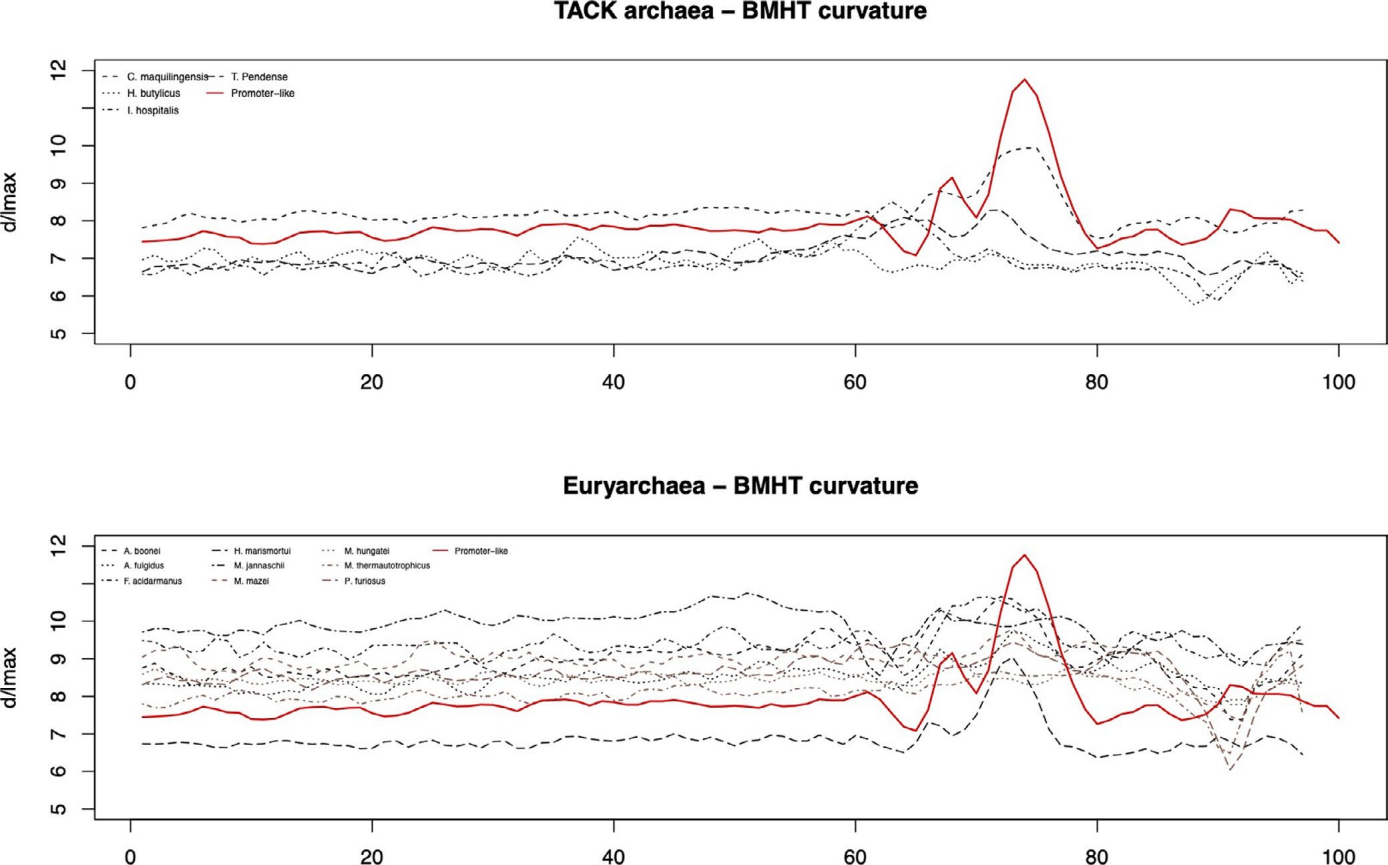
Bendability signal comparison of promoters and upstream regions of thirteen other archaea. The red line (promoter‐like) represents the average formed upon experimentally validated promoter of *H*. *volcanii*, *S*. *solfataricus*, and *T*. *kodakarensis* to be compared with upstream sequences of thirteen other archaea divided into two phylogenetic families. The remaining DDS, enthalpy, and BHMT curvature are found in Figures A2, A3, and A4, respectively

**FIGURE 7:**
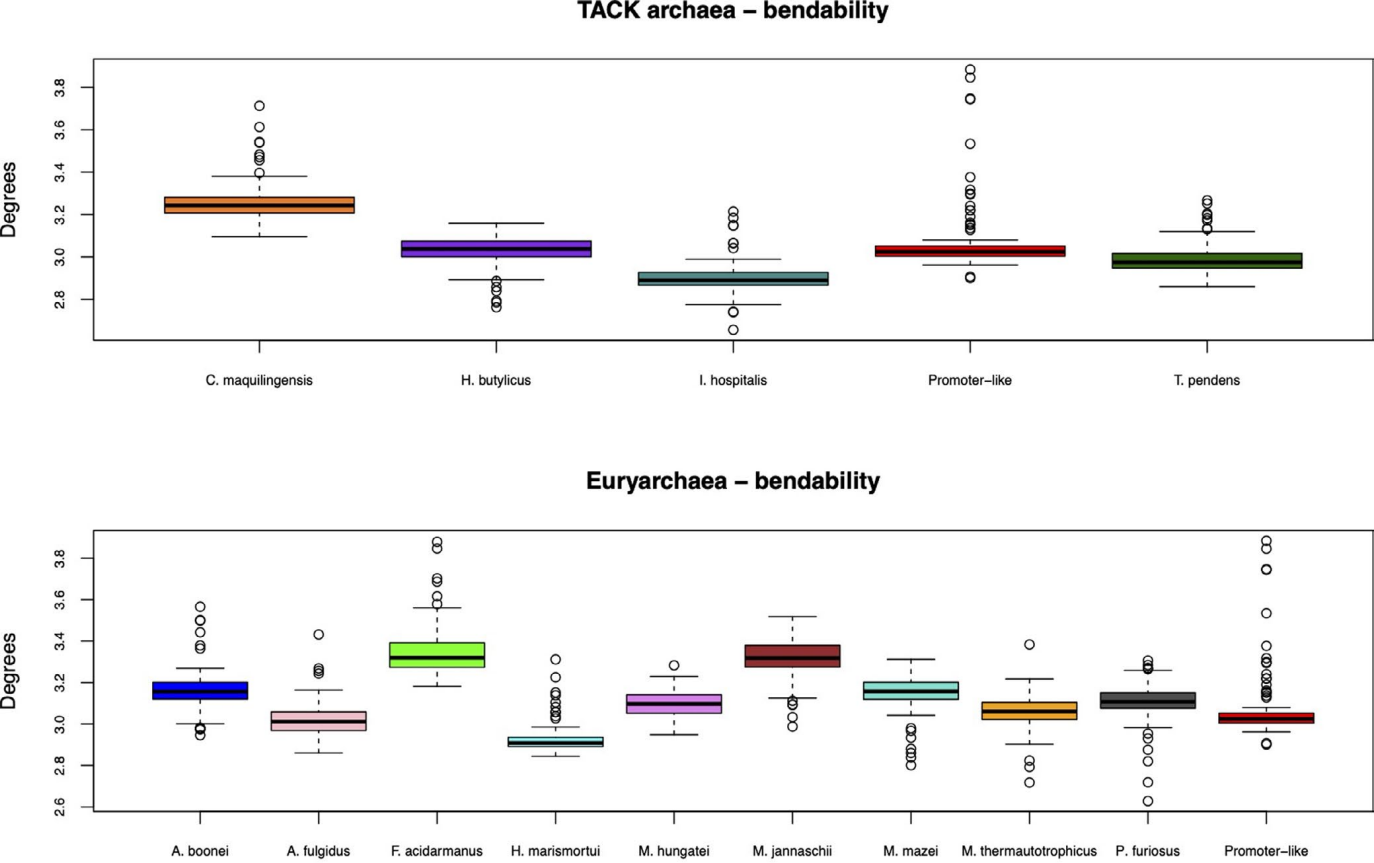
Boxplots of promoters and upstream regions of thirteen other archaea converted to bendability. The boxplots represent statistical comparisons between the promoter‐like profile, (red), formed upon experimental data of *H*. *volcanii*, *S*. *solfataricus*, and *T*. *kodakarensis*. The *p* < 2e‐16 values obtained by the nonparametric Kruskal–Wallis test conveyed statistical significance in the averages of both groups. Additional analyses encompassing BMHT curvature, enthalpy, and DDS are found in Figures A5, A6, and A7, respectively

**FIGURE A2:**
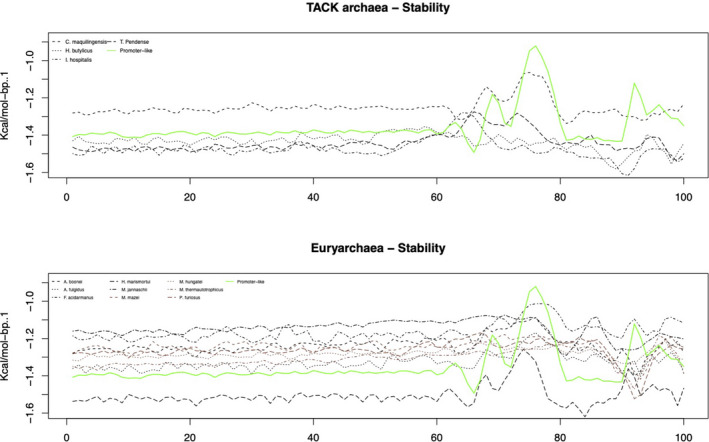
DNA Duplex Stability signal comparison of promoters and upstream regions of thirteen other archaea. The green line (promoter‐like) represents the average formed upon experimentally validated promoter of *H*. *volcanii*, *S*. *solfataricus*, and *T*. *kodakarensis* to be compared with upstream sequences of thirteen other archaea divided into two phylogenetic families

**FIGURE A3:**
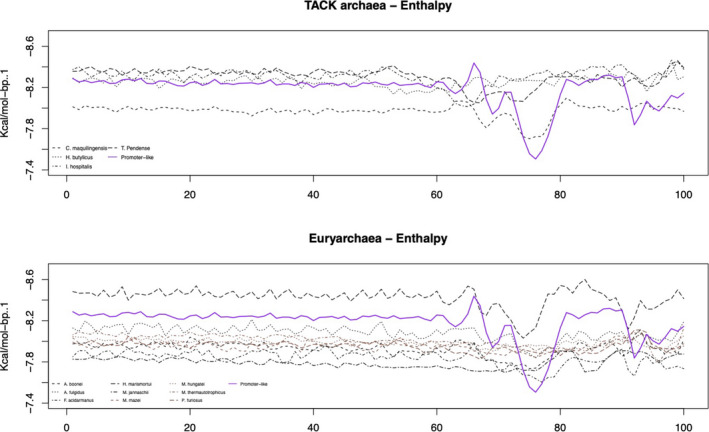
Enthalpy signal comparison of promoters and upstream regions of thirteen other archaea. The purple line (promoter‐like) represents the average formed upon experimentally validated promoter of *H*. *volcanii*, *S*. *solfataricus*, and *T*. *kodakarensis* to be compared with upstream sequences of thirteen other archaea divided into two phylogenetic families

**FIGURE A4:**
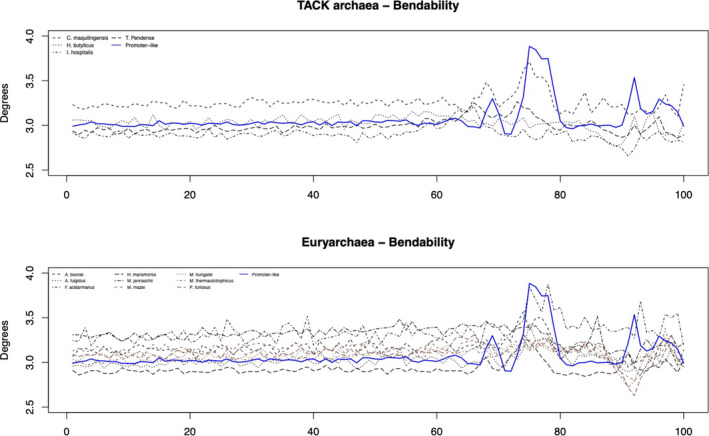
Bendability signal comparison of promoters and upstream regions of thirteen other archaea. The blue line (promoter‐like) represents the average formed upon experimentally validated promoter of *H*. *volcanii*, *S*. *solfataricus*, and *T*. *kodakarensis* to be compared with upstream sequences of thirteen other archaea divided into two phylogenetic families

Moreover, we included a comparison of the upstream regions found in 13 archaea against the promoter‐like profile established in 3.4. To perform a comparative analysis, the promoter‐like profile was compared with upstream regions of 13 other archaea split into their phylogenetic family (Figure 6). Since the profiles observed in Figure 6 are the same when another physical feature is tested, comparisons following DDS, enthalpy, and bendability are included in Figures A2, A3, and A4, respectively. Analysis of variance tests indicated each organism is significantly different than the other by presenting *p* < 2e‐16 in TACK archaea and *p* < 2e‐16 in *Euryarchaea*. The statistical analysis of the two archaeal families is visualized in boxplots available in Figure 7.

### 3.6 Conserved and degenerated TATA groups

**FIGURE 8:**
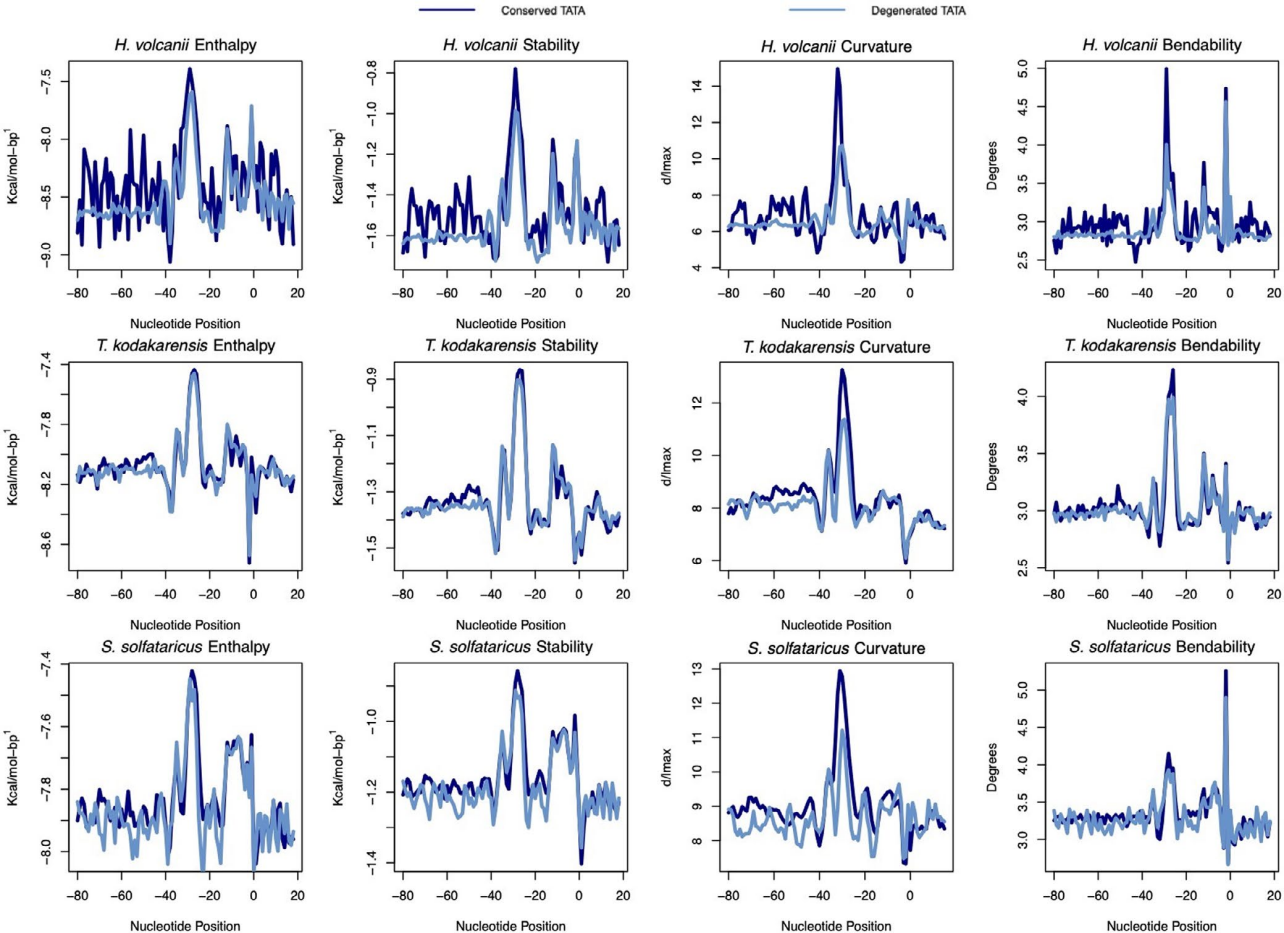
Structural/energetic profiles of conserved and degenerated TATA promoters. The conserved and degenerated TATA core promoter profiles are plotted. The lines represent the average value each group and organism showed. The navy‐blue lines represent sequences that had a MEME‐identified TATA motif, the light blue depicts sequences in which the specific TATA motif was not found

The core promoters belonging to conserved and degenerated TATA groups were converted into energetic and structural properties to indicate RNAP action in both groups (Figure 8). The two groups presented overlapping lines with strong signals being located around −28.

## 4 DISCUSSION

### 4.1 Nucleotide content

The results of this study suggest that TATA boxes slightly vary between organisms, supporting the archaeal diversification reported by (DeLong et al., 1994). Additionally, the AT content was found differently in each archaeon.

When the archaeal promoters were evaluated as owning either a conserved or a degenerated TATA consensus, the GC% of each organism has explained the conservation found upon TATA boxes, so the organism with higher genome GC% was the one that presented the least amount of TATAs, this is no news. However, the binding of TBP, TFB, and TFE to a TATA+BRE motif and TFE binding to PPE/INR were found through this *in silico* approach to be off from a primary sequence inspection, just as that conservation found around these motifs is not mandatory. Moreover, promoter activity is still observed when promoters lack a clear TATA motif (Aptekman & Nadra, 2018). Therefore, the uneven number of conserved TATA sequences sprung around archaea is explained by the dynamics of biology. The two groups of promoters (conserved and degenerated TATA) have also presented statistical significance in *H*. *volcanii* and *S*. *solfataricus* when the GC content was employed as a possible explanation for each group. This reassures the hypothesis that the probability of TATA boxes to be found depends directly on the genome composition of a given archaeon.

### 4.2 Energetic and structural parameters define promoter‐like profiles

Promoter sequences might be defined by a set of strong signals around their transcription factor binding sites (TFBS), that is, TFB, TBP, and TFE. In this study, the conversion of genetic information into physical attributes has protruded distinctive signals around TFBS of the proteins, while shuffled sequences did not. These strong signals are in favor of the relative location of the basal transcription factors (TF), which is explained by the laws ruling the promoter area. Both enthalpy and stability are energetic‐related features, the base pairs that are more commonly found in promoters are AT and their chemical conformation reflects in more energy available (Allawi & SantaLucia, 1997; de Avila e Silva et al, 2014; Privalov & Crane‐Robinson, 2018; SantaLucia & Hicks, 2004; Yella et al., 2018). The distinct signals represented by curvature and bendability are explained by the TFBS being more rigid and more curved, which acts against the formation of nucleosomes (Tirosh et al., 2007).

The profiles obtained in this study indicate a conserved aspect around the binding site of proteins that are key elements in the Pre‐Initiation Complex (PIC) formation. In vitro studies advocated for TBP+TFB being enough to begin transcription. Indeed, our results show conserved signals around this site (−27 2nt spacer −31). However, the inclusion of a signal in the vicinity of −10 and +1, which matches the TFE binding site, also contributes to promoter definition (Ao et al, 2013). This TF protein was reported to optimize the formation of PIC in TACK and other families as well (Hanzelka et al., 2001).

The signal located in the −10 region of three archaea is also an important factor in bacterial transcription (Lloréns‐Rico et al., 2015). Both bacteria and archaea share the same last unique common ancestor, and consequently, share similarities despite their evolution taking place in different branches of the tree of life (Gribaldo & Brochier‐Armanet, 2006).

The lack of annotation in the genome of many archaea creates the possibility for such methods. When the validation of the promoter identification method was tested in upstream regions of thirteen archaea, the same rationale was inferred. Mining published information upon transcripts has enabled the definition of a promoter‐like profile through a combination of strong signals in the binding sites of TBP, TFB, and TFE (−27, −31, −10, and +1, respectively). When data that do not encompass experimentally validated promoter sequences only was assessed, strong signals were observed in the ending of the sequences, suggesting that there might be promoter elements found in these intergenic areas, as identified by (Yella et al., 2018).

The observation of Figure 6 (and Figures A2, A3, and A4) assures the possibility of locating promoters in upstream regions due to their physical profile. Two archaea have shown TFBS signals similar to the promoter‐like profile: *A*. *boonei* and *T*. *pendens*. Even though there are differences in the signals protruded by promoters and potential promoters, resulting in significant differences between the groups’ averages, the second group poses for the rise of methods for promoter identification as the one brought by this study.

### 4.3 Promoter signal beyond TATA boxes

TATA boxes are likely the most conserved sites that distinguish both archaeal/eukaryotic promoters. The initiation of the transcription in archaea has been reported to start with TPB and TFB proteins attaching to the promoter (Gehring et al., 2016, Blombach & Grohmann, 2017) and enhanced by the presence of TFE (Hanzelka et al., 2001), this binding is assisted by the conservation found around the binding site of these proteins. Promoters have been grouped in terms of their TATA analysis in (Tirosh et al., 2007; Yella & Bansal, 2017), both authors performed structural conversions such as this study did. Divergent results could be observed in which TATA‐conserved sequences did not show significant differences when compared to TATA‐degenerated ones.

In this study, both TATA‐conserved and TATA‐degenerated groups have shown the same strong signals around the binding sites of TFB, TBP, and TFE. Some differences might protrude mathematical variance, for example, the TFB and TBP binding sites analyzed in the curvature profile of three archaea and *H*. *volcanii* bendability and DDS. This feature defines the promoter (either TATA‐conserved or not) as a promoter‐like sequence, which is a novel approach in identifying and finding new promoter sequences in archaea.

## 5 CONCLUSIONS

The results we demonstrated in this study encourage the DNA codification into energetic/structural attributes that reveal transcription factor proteins binding sites where a primary sequence inspection failed. Hence, this study poses a novel method to be used in genome annotation regarding archaeal promoters.

## CONFLICT OF INTERESTS

None declared.

## AUTHOR CONTRIBUTIONS

**Gustavo Sganzerla Martinez:** Conceptualization (equal); Data curation (equal); Formal analysis (equal); Funding acquisition (equal); Investigation (equal); Methodology (equal); Project administration (equal); Resources (equal); Software (equal); Supervision (equal); Validation (equal); Visualization (equal); Writing‐original draft (equal); Writing‐review & editing (equal). **Sharmilee Sarkar:** Conceptualization (equal); Data curation (equal); Formal analysis (equal); Funding acquisition (equal); Investigation (equal); Methodology (equal); Project administration (equal); Resources (equal); Software (equal); Supervision (equal); Validation (equal); Visualization (equal); Writing‐original draft (equal); Writing‐review & editing (equal). **Aditya Kumar:** Conceptualization (equal); Data curation (equal); Formal analysis (equal); Funding acquisition (equal); Investigation (equal); Methodology (equal); Project administration (equal); Resources (equal); Software (equal); Supervision (equal); Validation (equal); Visualization (equal); Writing‐original draft (equal); Writing‐review & editing (equal). **Scheila de Avila e Silva:** Conceptualization (equal); Data curation (equal); Formal analysis (equal); Funding acquisition (equal); Investigation (equal); Methodology (equal); Project administration (equal); Resources (equal); Software (equal); Supervision (equal); Validation (equal); Visualization (equal); Writing‐original draft (equal); Writing‐review & editing (equal). **Ernesto Perez‐Rueda:** Conceptualization (equal); Data curation (equal); Formal analysis (equal); Funding acquisition (equal); Investigation (equal); Methodology (equal); Project administration (equal); Resources (equal); Software (equal); Supervision (equal); Validation (equal); Visualization (equal); Writing‐original draft (equal); Writing‐review & editing (equal).

## ETHICS STATEMENT

None required.

## Figures and Tables

**FIGURE A5:**
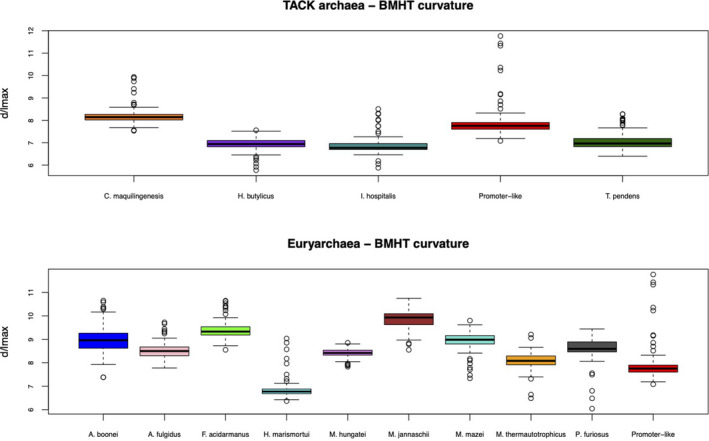
Boxplots of promoters and upstream regions of thirteen other archaea converted to BMHT curvature. The boxplots represent statistical comparisons between the promoter‐like profile, (red), formed upon experimental data of *H*. *volcanii*, *S*. *solfataricus*, and *T*. *kodakarensis*. The *p* < 2e‐16 values obtained by the nonparametric Kruskal–Wallis test conveyed statistical significance in the averages of both groups

**FIGURE A6:**
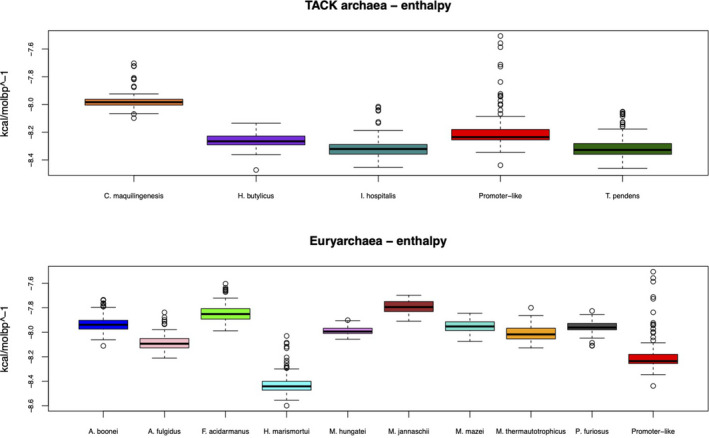
Boxplots of promoters and upstream regions of thirteen other archaea converted to enthalpy. The boxplots represent statistical comparisons between the promoter‐like profile, (red), formed upon experimental data of *H*. *volcanii*, *S*. *solfataricus*, and *T*. *kodakarensis*. The *p* < 2e‐16 values obtained by the nonparametric Kruskal–Wallis test conveyed statistical significance in the averages of both groups

**FIGURE A7:**
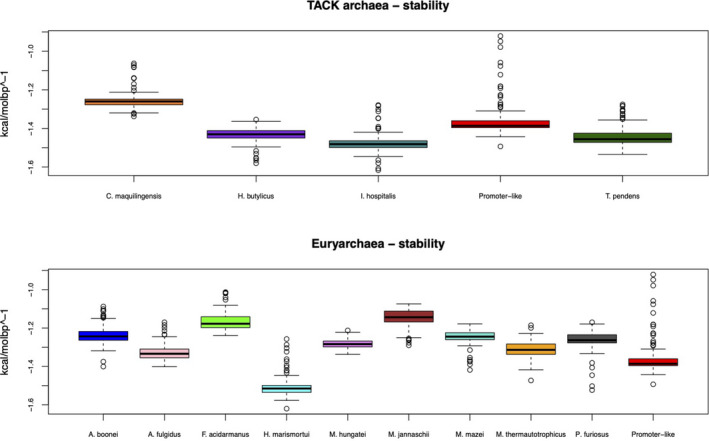
Boxplots of promoters and upstream regions of thirteen other archaea converted to stability. The boxplots represent statistical comparisons between the promoter‐like profile, (red), formed upon experimental data of *H*. *volcanii*, *S*. *solfataricus*, and *T*. *kodakarensis*. The *p* < 2e‐16 values obtained by the nonparametric Kruskal–Wallis test conveyed statistical significance in the averages of both groups

## Data Availability

The experimentally verified promoter sequences were retrieved from their original publications: H. volcanii (https://doi.org/10.1186/s12864‐016‐2920‐y), S. solfataricus (https://doi.org/10.1101/gr.100396.109), and T. kodakarensis (https://doi.org/10.1016/0022‐2836(91)90492‐O). The upstream regions used in the method validation step were extracted from RSAT Database (http://www.rsat.eu). The supplementary material is available in the Zenodo repository: (1) the sequence IDs and the gene annotation of all H. volcanii, T. kodakarensis, and S. solfataricus promoters used in this study: https://doi.org/10.5281/zenodo.5137550, (2) the Python scripts S1–S4 employed in the structural analysis of archaeal promoter sequences: https://doi.org/10.5281/zenodo.5137597
